# Depression Amplifies the Influence of Central Obesity on 10-Year Incidence of Diabetes: Findings from MIDUS

**DOI:** 10.1371/journal.pone.0164802

**Published:** 2016-10-18

**Authors:** Vera K. Tsenkova, Arun Karlamangla

**Affiliations:** 1 University of Wisconsin, Madison, Institute on Aging, Madison, Wisconsin, United States of America; 2 UCLA, Division of Geriatrics, Los Angeles, California, United States of America; Baylor College of Medicine, UNITED STATES

## Abstract

**Background:**

Central obesity is a major risk factor for diabetes but many obese individuals never develop diabetes, suggesting the presence of important effect modifiers. Depression has emerged as a key risk factor for poor glycemic control, but to our knowledge, no previous work has investigated whether depression amplifies the effect of central obesity on glucoregulation.

**Methods and Findings:**

We used a national sample of adults without prevalent diabetes (MIDUS; N = 919) to test for synergy between central obesity and depression in the development of diabetes 10 years later. We found that depression amplified the association of waist-to-hip ratio (WHR) with incident diabetes adjusted for age, race, gender, education, physical activity, and sleep problems (*p* = 0.01 for test of interaction). The relative risk for incident diabetes per every 0.1 increment in WHR was 1.75 (95% CI: 1.31; 2.33) in those without depression and 3.78 in those with depression (95% CI: 2.14; 6.66).

**Conclusions:**

These results confirm the role of depression as a robust risk factor for the development of diabetes and for the first time, demonstrate a synergy between depression and central obesity. Identifying and addressing depression could prove to be an effective approach to preventing diabetes in at risk individuals. Ultimately, elucidating the interplay among risk factors from different domains will be key to understanding multifactorial diseases such as diabetes and informing theory-based, patient-centered interventions aimed at reducing diabetes risk.

## Introduction

Type 2 diabetes mellitus is increasing in prevalence in both developed and developing economies, and is a growing public health crisis that is responsible for substantial morbidity and premature mortality worldwide. According to the National Diabetes Statistics Report [1], 9.3% of the U.S. population (29.1 million people) have diabetes and 86 million people have prediabetes. Diabetes imposes a substantial burden on U.S. economy: the total cost of diagnosed diabetes in 2012 was $245 billion and care for people with diabetes accounted for more than 1 in 5 healthcare dollars [2].

An emerging line of research has identified depression as a key risk factor for type 2 diabetes. Depressed adults have 37% to 60% higher risk of developing diabetes than adults without depression [3,4]. Depressive symptoms have also been linked to metabolic abnormalities which precede the development of diabetes [5] as well as poor prognosis [6,7]. Although the direction of the relationship between depression and glucose metabolism has been questioned [8], behavioral and physiological mechanisms have been proposed as explanations for the increased risk of diabetes among depressed adults. For example, depressed individuals show abnormalities in the hypothalamic-pituitary-adrenal axis, particularly in regulation of cortisol, which is in turn related to dysregulated glucose metabolism [9–11]. Importantly, depression is associated with obesity-promoting behaviors [12,13], and there is little doubt that the current epidemic of type 2 diabetes has been fueled by the recent rise in obesity levels. Central obesity in particular has been identified as a key culprit, as visceral adipose tissue is known to generate diabetogenic substances [14]. However, while more than 80% of people with type 2 diabetes are obese, most obese people never develop diabetes [15], suggesting that there are hitherto unrecognized modifiers of the obesity-conferred diabetes risk. Obesity and depression share underlying pathophysiological mechanisms (e.g., low grade inflammation) [16], and we investigated whether the co-occurrence of both amplified the risk of developing diabetes. To our knowledge, no previous study has investigated whether depression and central obesity synergistically influence glucose metabolism.

We use longitudinal data from the Midlife Development in the U.S. (MIDUS) national study to investigate potential synergies between depression and central obesity on glucoregulation, vis-à-vis both incident diabetes and levels (severity) of glucose dysregulation across pre-disease and clinical disease states. We build on prior work to address two key questions: a) Is depression related to the incidence of diabetes over the 10-year period between the 2 waves of MIDUS data collection and the levels of glucose dysregulation in wave 2? and b) Does depression exacerbate the causal link between central obesity and incident diabetes and glucose dysregulation?

## Methods

### Sample

Data were drawn from MIDUS, a longitudinal study of health and well-being. MIDUS 1 began in 1995–96 as a national random digit dial sample of non-institutionalized, English-speaking adults living in the United States. A final sample of 7108 participants ages 25–74 completed telephone and mail surveys in MIDUS 1. Approximately 9–10 years later, 4963 (75% response rate adjusted for mortality) were successfully contacted to participate in another phone interview and self-administered questionnaire (MIDUS 2 Survey). Participants who completed both MIDUS 1 and MIDUS 2 Survey were invited to be part of the MIDUS biomarker project. Participants who were healthy enough to travel and consented to participate in the biomarker project were invited to stay overnight at one of three regional General Clinical Research Centers (GCRCs) at UCLA, Georgetown, or the University of Wisconsin. MIDUS 1 was approved by the institutional review board (IRB) at Harvard and MIDUS 2 was approved by the IRBs at each GCRC (Georgetown University IRB, UCLA IRB, University of Wisconsin Health Sciences IRB), and informed written consent was obtained from all participants. Further details of the study design, recruitment, and retention are available at http://www.icpsr.umich.edu/icpsrweb/NACDA/. The current study used data from MIDUS 1 and MIDUS 2. Of the 1054 participants from the MIDUS 1 cohort who participated in the biological data collection in MIDUS 2, 135 cases were excluded due to partially missing data on any variable in the analysis (N = 115) or reporting diabetes at baseline (N = 20), resulting in 919 participants with complete data.

### Measures

The outcome variables were measured in MIDUS 2. Diabetes was defined using current criteria from the American Diabetes Association: HbA_1c_ ≥ 6.5% or fasting glucose ≥126 mg/dl, or taking medications that lower glucose [17]. Fasting glucose and HbA_1c_ samples were obtained during an overnight stay in a GCRC. Participants were instructed to bring all their medications to the GCRC stay and project staff recorded medication names and other relevant information. Since no participants had diabetes in MIDUS 1, the presence of diabetes in MIDUS 2 reflected incidence of diabetes over the 9–10 year period between the two waves of data collection.

In order to explore glucoregulation at a more granular level that span from pre-disease to clinical glycemia, we created a glucose dysregulation index, a 4-level ordinal outcome that differentiated between normoglycemia, pre-diabetes, diabetes without pharmacological treatment, and diabetes with pharmacological treatment. Prediabetes was defined as HbA_1c_ 5.7–6.4% or fasting glucose 100–125 mg/dl, and NOT taking diabetes medications.

All independent variables were measured in MIDUS 1. Depression diagnosis was coded as a binary variable and assessed with information from the phone interview and defined according to criteria in the third edition of the Diagnostic and Statistical Manual of Mental Disorders (DSM-III-R) [18]. A diagnosis of past year depressive episode required that a person experienced a period of at least two weeks of either depressed mood or anhedonia most of the day, nearly every day, and a series of at least four symptoms typically found to accompany depression, such as problems with eating, sleeping, energy, concentration, feelings of self-worth, and suicidal thoughts or actions [19]. Waist circumference at the level of the navel and hip circumference at the widest point were self-reported (in inches) by respondents using a tape measure and diagram provided by the MIDUS staff. Waist-to-hip ratio (WHR) was calculated by dividing waist circumference by hip circumference.

Demographic covariates included age (in years), gender (male or female), and race/ethnicity (white or Minority). Participants reported their highest level of education and a 12-point scale was constructed, ranging from 1 (no schooling or some grade school) to 12 (professional degrees such as PhD or MD). Health behaviors included physical activity and sleep problems. Physical activity was assessed by two questions (one for each season): “During the winter/summer, how often do you engage in vigorous physical activity (e.g., running or lifting heavy objects) long enough to work up a sweat?” Response choices were “several times a week or more”, “about once a week”, “several times a month”, “about once a month”, “less than once a month”, and “never”. The responses to each question were coded so that they represented estimated numbers of exercise sessions per month and the two estimates were averaged to create a measure of vigorous activity. A dummy code was created for sleep problems if respondent responded yes to question “In the past twelve months, have you experienced or been treated for chronic sleeping problems.”

### Statistical Analyses

Modified Poisson regression with robust error estimation was used to model the associations of depression (yes/no) and WHR (continuous) in MIDUS 1 with the two MIDUS 2 outcomes: overt diabetes (yes/no) or glucose dysregulation (ordinal; 0 to 3). Modified Poisson regression with robust error estimation is the preferred method for modeling binary outcomes that are not rare such as the diabetes outcome in our study [20]. WHR was treated as a continuous predictor because an examination of incident diabetes as a function of WHR deciles with and without adjusting for age and age squared showed a linear trend across the entire range of WHR.

The main effects of WHR and depression on incident diabetes and glucose dysregulation were examined with mutual adjustment and controlled for relevant covariates: age, age squared, race, gender, education categories, vigorous physical activity, sleep problems, and an interaction between education and gender. Models testing for an interactive effect between depression and WHR on incident diabetes and glucose dysregulation further controlled for interactions between the primary predictors and age andgender.

## Results

Descriptive data for all independent variables measured in MIDUS 1 and glucose regulation outcomes measured in MIDUS 2 are presented in Table 1 for the full sample (N = 919) and stratified by diabetes status at MIDUS 2. On average, participants in the current study were aged 46 years at baseline, 93% were white, and 55% were women. Depression was present in 122 participants in the study sample (13%). Participants reported exercising on average 7 times a month. Participants who developed diabetes were older (*p* < .001), had higher WHR (*p* < .001), exercised less (*p* < .05), and were less educated (*p* < .05) at baseline than participants who did not develop diabetes. Further, participants who developed diabetes were less likely to be white (*p* < .01) and marginally more likely to be male (*p* = .06). There were no significant differences in depression prevalence among participants with diabetes and participants without diabetes (*p* = .83). The 10-year incidence of diabetes was 13% (n = 115). In MIDUS 2, an additional 444 participants (48%) had pre-diabetes, and 360 participants (39%) had normal glucoregulation.

**Table 1:** Descriptive Statistics.

	No Diabetes in MIDUS 2	Diabetes in MIDUS 2	Total MIDUS 2 Sample	
	(N = 804)	(N = 115)	(N = 919)	
	Mean (*SD*) or %	Mean (*SD*) or %	Mean (*SD*) or %	Range
**MIDUS 1 Predictors**				
Depression				0–1
Yes	13%	14%	13%	
No	87%	86%	87%	
Waist to Hip Ratio	.87 (.10)	.92 (.09)	.87 (.10)	.55–1.15
Race				0–1
White	94%	87%	93%	
Other	6%	13%	7%	
Age	45.3 (11.6)	51.9 (11.8)	46.1 (11.8)	25–74
Gender				0–1
Female	56%	47%	55%	
Male	44%	53%	45%	
Education	7.6 (2.4)	7.1 (2.4)	7.6 (2.4)	2–12
Exercise	6.9 (5.2)	5.3 (5)	6.7 (5.2)	0–13
Sleep Problems				0–1
Yes	10%	14%	11%	
No	90%	86%	89%	
**MIDUS 2 Outcomes**				
Normoglycemia			39%	
Prediabetes			48%	
Diabetes, No Meds			6%	
Diabetes, On Meds			7%	

**Fig 1:**
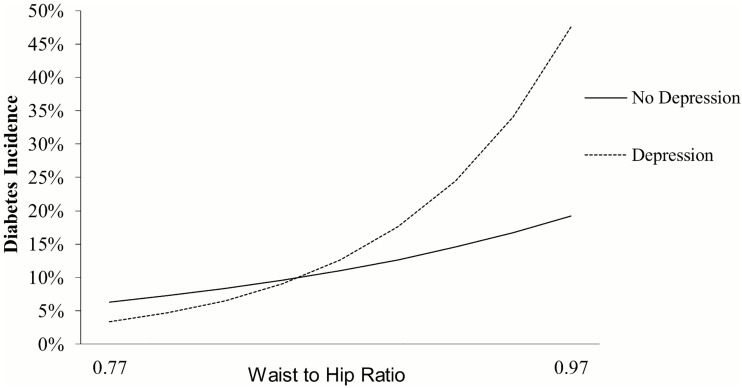
Model-predicted diabetes incidence risk as function of baseline depression (yes/no) and waist-to-hip ratio.

Poisson regression models predicting diabetes incidence are presented in Models 1.1 and 1.2 (Table 2). As expected, larger WHR was associated with higher diabetes incidence: the adjusted relative risk per standard deviation (SD, or 0.1) increment in WHR was 1.76 (95% CI: 1.42; 2.18; Model 1.1). Depression was not significantly associated with diabetes risk in main effect models (*p* value for depression = 0.6; Model 1.1). However, there was a significant interaction between WHR and depression, such that depression amplified the influence of WHR on diabetes incidence 10 years later (See Model 1.2; *p* value for interaction = 0.01). As a result, the relative risk for incident diabetes per every 0.1 increment in WHR was 1.75 (95% CI: 1.31; 2.33) in those without depression and 3.78 in those with depression (95% CI: 2.14; 6.66). Fig 1 illustrates the synergistic relationship between WHR and depression on diabetes incidence. Simple slope analyses on Model 1.2 showed that although there was no association between depression and diabetes incidence at average values of WHR, there was a detrimental association at higher levels of WHR: at 0.97 (which was 1 SD above the mean), relative risk was 2.48 (95% CI: 1.10; 5.59) and at 1.07 (or 2 SD above the mean), relative risk was 5.36 (95% CI: 1.50; 19.12). While others have suggested that women show increased risk for diabetes at lower WHR levels than men [21], we found no evidence that the association between WHR and diabetes incidence depended on age or gender (interaction *p* values >.2).

**Table 2:** Waist-to-hip ratio (WHR) and Depression Predict Incident Diabetes and Level of Glucose Dysregulation.

	*Incident Diabetes Risk Ratio (95% CI)*		*Level of Glucose Dysregulation Rate Ratio (95% CI)*	
Predictors	Model 1.1 ^a^	Model 1.2^b^	Model 2.1 ^a^	Model 2.2 ^b^
WHR	*1*.76***	*1*.75***	*1*.28***	*1*.27***
	*(1*.*42; 2*.*18)*	*(1*.*31; 2*.*33)*	*(1*.*17; 1*.*39)*	*(1*.*13; 1*.*42)*
Depression	*1*.*13*	*1*.*14*	*1*.*18*^≠^	*1*.35*
	*(*.*7; 1*.*83)*	*(*.*59; 2*.*24)*	*(*.*99; 1*.*41)*	*(1*.*05; 1*.*73)*
WHR x Depression		*2*.16*		*1*.24*
		*(1*.*18; 3*.*98)*		*(1*.*01; 1*.*53)*

^*≠*^ *p <* .*075*

* *p* < .05.

** *p* < .01.

***p < .001.

Note

^a^. Models 1.1 and 2.1 adjust for Age (continuous, linear and squared terms), Race, Gender, Education, Physical Activity, and Sleep Problems, and the following interaction: Education X Gender. The reported associations for WHR are per 1 SD increment.

^b^ Models 1.2 and 2.2 include all terms from ^a^ plus the following interactions: WHR x Depression, Depression x Gender, WHR x Gender, Depression x Age, and WHR x Age. In these models, the main effect of depression is the effect of depression at the mean level of WHR. The reported associations for WHR are per 1 SD increment.

**Fig 2:**
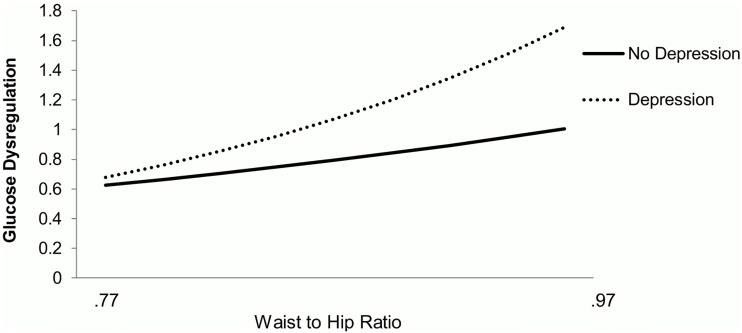
Model-predicted level of glucose dysregulation levels 10 years later as function of baseline depression (yes/no) and waist-to-hip ratio. Legend: The effect size reported is the rate ratio at which an individual moves up a level of glucose dysregulation.

Poisson regression models predicting level of glucose dysregulation are presented in Models 2.1 and 2.2; the reported effect size is the rate ratio–the ratio of the rate at which an individual moves up a level of dysregulation (Table 2). Larger WHR was associated with higher levels of glucose dysregulation: the adjusted rate ratio per SD increment in WHR was 1.28 (95% CI: 1.17; 1.39; Model 2.1). Depression was not significantly associated with glucose dysregulation level (*p* = .06). Similarly to the models predicting 10-year diabetes incidence, there was a significant interaction between WHR and depression, such that depression amplified the influence of WHR on level of glucose dysregulation (See Model 2.2 and Fig 2; *p* value for interaction = 0.04). As a result, the rate ratio for glucose dysregulation per every 0.1 increment in WHR was 1.27 (95% CI: 1.13; 1.42) in those without depression and 1.57 in those with depression (95% CI:1.30; 1.92). Simple slope analyses on Model 2.2 documented a detrimental association at higher levels of WHR: at 1 SD above mean, the rate ratio was 1.68 (95% CI: 1.12; 2.51) and at 2 SD above mean, the rate ratio was 2.09 (95% CI: 1.16; 3.77). The association between WHR and diabetes incidence did not depend on age or gender (interaction *p* values>.1).

## Discussion

Type 2 diabetes is a multifactorial disease affected by the interplay of biological, genetic, sociodemographic, and psychosocial influences. More than one third of American adults were obese in 2009–2010, up from a mere 5–6% only three decades ago [22], and obesity, especially central obesity, is the major risk factor for development of type 2 diabetes. We examined the prospective associations of central obesity and depression with glucose metabolism 10 years later among adults without diabetes at baseline, and documented a synergy between depression and central obesity.

The co-occurrence of depression with metabolic risk factors (e.g., high triglycerides, hypertension, and abdominal obesity) has been labeled ‘metabolic depression’ [23,24] and it has been suggested that the combination of depression and metabolic dysregulation is the fundamental risk factor associated with increased diabetes risk [25]. Our key finding that depression amplified the obesity-conferred risk of developing diabetes supported this idea and is consistent with a recent study that documented a synergistic interaction between depressive symptoms and metabolic dysregulation on type 2 diabetes incidence [25] and with prior findings that some negative psychological states amplify the detrimental influence of obesity on glucose metabolism [26–31]. Adjustments for demographics, socioeconomic status, and health status and behaviors were included in our analyses, thereby sharpening the focus on the distinctive contribution of depression and central obesity to glycemic control. Hypercortisolemia is a likely mechanism underlying the observed depression-central obesity synergy, as cortisol can interfere in glucose regulation by altering both insulin secretion and insulin sensitivity [32]. Depression is associated with HPA axis dysregulation in the form of increased cortisol secretion and altered diurnal cortisol rhythm, and central adipose tissue is rich in glucocorticoid receptors. If indeed insulin resistance is the result of the metabolic effects of products released by central adipose tissue [33,34], then higher circulating levels of cortisol in depressed individuals could exacerbate obesity-associated glucose dysregulation [35].

Our findings should be interpreted in light of study limitations. A key limitation of this study is the lack of biological data at baseline (MIDUS 1): although we excluded participants with self-reported diabetes at MIDUS 1, we may have failed to exclude a few individuals with undiagnosed diabetes. This is unlikely to be a source of significant bias in our results: even if a third of the people with diabetes at baseline had undiagnosed diabetes and were included in our analyses, we would have failed to exclude 10 people of the 115 who had diabetes at follow-up. Further, our analyses were modeled to capture risk for developing type 2 diabetes, but information was not available on whether participants had type 1 or type 2 diabetes. Given that only 5–10% of Americans with diabetes have type 1 diabetes [17], and that the incidence of type 1 diabetes in adults is even smaller, our results are unlikely to have been significantly affected by this misclassification. Finally, WHR was self-reported, which is generally plagued by under-reporting. Our sample was comprised primarily of white participants and it is important for future research to investigate whether our findings generalize to minority groups for whom the prevalence and incidence of obesity, depression, and diabetes may differ. Notable strengths of our study include the national sample, sample size, length of follow up, ascertainment of incident diabetes using both fasting glucose and HbA1c measurements, and examining glucose dysregulation across the range of the glycemic continuum.

Our findings underscore the importance of assessing mental health and psychological well-being in patients at risk for diabetes. Patients and providers will benefit from recognizing that depression strongly predicts glucose dysregulation and increased diabetes risk, and that the impact of depression is accentuated in people with central obesity. While the importance of promoting physical activity and weight loss in people at risk for diabetes is undisputed, identifying and addressing depression could prove to be an equally important and effective approach to preventing diabetes. Future research will need to test the efficacy of interventions that promote psychological well-being and/or treat depression in preventing the development of diabetes in high-risk individuals.
